# Concurrent sensorimotor temporal recalibration to different lags for the left and right hand

**DOI:** 10.3389/fpsyg.2014.00140

**Published:** 2014-02-25

**Authors:** Yoshimori Sugano, Mirjam Keetels, Jean Vroomen

**Affiliations:** ^1^Department of Industrial Management, Kyushu Sangyo UniversityFukuoka, Japan; ^2^Department of Cognitive Neuropsychology, Tilburg UniversityTilburg, Netherlands

**Keywords:** adaptation, temporal recalibration, motor-sensory synchrony, tapping, sensorimotor coordination, delayed auditory feedback, internal clock

## Abstract

Perception of temporal synchrony between one’s own action and the sensory feedback of that action is quite flexible. We examined whether sensorimotor temporal recalibration (TR) involves central or motor-specific components by concurrently exposing the left and right hands to different lags. The experiment was composed of a pre-test, an adaptation phase, and a post-test. During the adaptation phase, participants tapped their left and right index fingers in alternating fashion while each tap induced an auditory feedback signal (a short click sound). One hand was exposed to a long delay between the tap and the sound (~150 ms), while the other hand was exposed to a subjective no-delay (~50 ms). Before and after the adaptation phase (the pre- and post-test), participants tried to tap in synchrony with pacer tones (ISI = 1000 ms). The results showed that the hand that was exposed to the delayed sound corrected for this delay by tapping earlier (a larger anticipation error) than the no-delay hand, indicating TR. Different amounts of TR were found when the left and right hand were concurrently exposed to the same versus different delays. With different exposure- delays for the two hands, there was a TR even for the hand that did not experience any delay in the feedback signal. However, it is not the case with the same exposure delay for the two hands. TR of the hand that experienced delayed feedback also occurred faster and was more complete (~40% greater than that of the hand with no subjective delay) if the two hands were exposed to the same rather than different delays (~20% greater than that of the hand with no subjective delay). These results suggest the existence of cross-talk between the hands, where both central and motor-specific components might be involved.

## INTRODUCTION

Perception of temporal synchrony between one’s own action (e.g., tapping) and a sensory feedback following the action (e.g., a flash or a tone) can be flexibly changed after prolonged exposure of an artificially induced temporal delay of the sensory feedback, which sometimes leads to a reversed sensation of the cause-effect relationship (Cunningham et al., 2001; Stetson et al., 2006; Heron et al., 2009; Sugano et al., 2010, 2012; Stekelenburg et al., 2011; Keetels and Vroomen, 2012). This remarkable flexibility of sensorimotor timing is often explained by the concept of temporal recalibration (TR; Fujisaki et al., 2004; Vroomen et al., 2004). However, the mechanism underlying sensorimotor TR is still unclear (for review, see Vroomen and Keetels, 2010). One plausible hypothesis for sensorimotor TR is that a single supramodal mechanism, which is usually referred to as a “central clock,” is responsible for adapting to the perceived time across all sensory pairings, including motor timing. This central clock refers to a single, dedicated centralized internal-time-keeper mechanism in which pulses are generated by a pacemaker and are counted by a counter (Creelman, 1962; Treisman, 1963). This idea is in line with data showing equal amounts of sensory TR across all sensory pairings (Hanson et al., 2008). Support for this concept also comes from studies showing that sensorimotor TR readily transfers between sensory modalities (Heron et al., 2009; Sugano et al., 2010), and transfers from learned to novel tasks (Fujisaki et al., 2004; Pesavento and Schlag, 2006).

However, there is other evidence that is difficult to reconcile with a centralized-clock model. Instead, this evidence points toward early, peripheral timing mechanisms that are selective for modality and low-level stimulus features (for review, see Eagleman, 2008). For example, some researchers reported a complete absence of recalibration outside the audio-visual domain (Navarra et al., 2007; Harrar and Harris, 2008), while others reported relatively lower levels of a visuo-tactile recalibration mechanism that operates separately for the left and right hand (Takahashi et al., 2008). The magnitude of audio-motor recalibration has also been found to be greater than visual-motor and tactile-motor recalibration, and there are also costs involved when the modality of the sensory event changes between the adaptation phase and test phase (Heron et al., 2009). Moreover, it has been reported that audio-motor recalibration does not transfer to visuo-motor synchronization tasks (Sugano et al., 2012). Training in a visual temporal order judgment (TOJ) task also does not transfer to an auditory TOJ task and vice versa (Alais and Cass, 2010). Furthermore, training on auditory interval discrimination does not transfer to visual interval discrimination (Lapid et al., 2009; Grondin and Ulrich, 2011). It has been demonstrated that when presenting a beep and flash coming from a single location after a voluntary action with variable delays, the motor-auditory timing was recalibrated independently from the motor-visual timing (Parsons et al., 2013).

Striking evidence against the notion of a central clock involves concurrent recalibration in audio-visual synchrony perception (Roseboom and Arnold, 2011; Heron et al., 2012; Yuan et al., 2012). Here, it has been reported that observers can have multiple concurrent estimates of audio-visual synchrony for different audio-visual pairings, and TR can occur in positive and negative directions concurrently, provided that the signals are spatially or contextually separated.

However, it is unclear if such concurrent recalibration is possible for domains other than audio-visual temporal processing. It is of special interest if concurrent TR occurs for sensorimotor synchronization, because in the sensorimotor domain the perceived delay between an action and its consequence can be diminished due to intentional binding (Haggard et al., 2002). Some studies have indeed suggested that separate multiple-clocks exist in sensorimotor temporal processing (e.g., Parsons et al., 2013; Yarrow et al., 2013). Yarrow et al. (2013) compared within- and across-limb transfer of sensorimotor TR and suggested that the former reflected a genuine shift in neural timing (peripheral mechanism), while the latter was achieved via a criterion shift (central mechanism), suggesting the existence of separate peripheral timing mechanisms between limbs. Parsons et al. (2013) have shown that independent shifts of timing in response to an auditory and a visual stimulus occur when they are presented with different delays after a motor action, suggesting multiple independent timelines coexisting within the brain. Moreover, it has been shown that patients with a unilateral deficit in the cerebellum showed more variable tapping with their hand and foot corresponding to the impaired side. However, such variability is not observed in the case of the effectors corresponding to the contra-lateral side (Ivry et al., 1988). This observation also indicates that there can be separate timing systems for the two sides of the body (Ivry and Richardson, 2002).

Though these studies offer support for a multiple-clock model in controlling sensorimotor coordination, the concept has not been directly tested in the context of concurrent adaptation. Here, we therefore have sought to verify whether or not concurrent sensorimotor TR occurs for the left and right hand after exposure to different lags. We used motor-auditory pairings rather than motor-visual pairing since the former is expected to evoke greater effects (Heron et al., 2009; Sugano et al., 2012).

### PREDICTIONS

**FIGURE 1:**
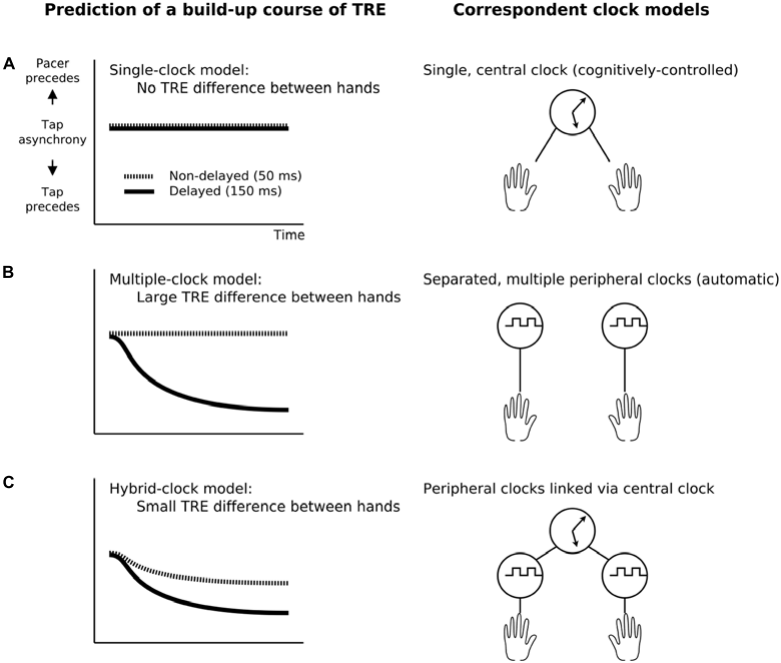
**Predictions about the build-up course of the tap asynchronies according to the three models of the internal clock.** The temporal recalibration effect (TRE) was defined as the change in tap-asynchrony between the pre- and the post-test. **(A)** The single clock model assumes a single clock regulating all kinds of temporal information in the brain, predicting no TRE difference between the left and right hands as they are completely pooled with each other. **(B)** The multiple-clock model assumes peripherally localized different clocks, predicting a TRE difference between hands as the hands are adapted separately via each clock. **(C)** The hybrid-clock model assumes both a central and peripheral clocks which are linked toghether, predicting smaller difference of the TRE than the multiple-clock model as the hands are adapted separately via peripheral clocks but cross-talked via a central one.

We hypothesized three possible models for temporal control mechanisms that might explain multiple concurrent TR for different sensorimotor delays: a single-central-clock model, a multiple-peripheral-clock model, and a hybrid-clock model (single-central plus multiple-peripheral clocks). Predictions generated by these three models are shown in **Figure 1**.

We predicted that TR in a tapping task, in which participants try to tap in synchrony with an auditory pacing signal, will manifest itself as a compensatory shift in the natural negative asynchrony between the tap and pacing signal. After exposure to delayed sensory feedback, observers thus were expected to tap *earlier* to compensate the previously experienced delay (Sugano et al., 2012). The rationale for this is from the Paillard–Fraisse hypothesis and its modified version, the sensory accumulator model (e.g., Aschersleben and Prinz, 1997; Aschersleben et al., 2001; Aschersleben, 2002). This model assumes that the perceived timing of a pacing signal and the perceived timing of a tap should be synchronized at the level of central representations in a synchronization task and the difference of perceptual latencies between them causes the tap-asynchrony.

The single-central-clock model assumes that a single, unified (e.g., amodal) clock regulates all temporal coordination in the brain. It predicts that tap asynchronies do not differ between the left and right hands if they were exposed to different delays, because the effects of lag adaptation for the left and right hand are “pooled” together via a single central mechanism (**Figure 1A**). In contrast, the multiple-peripheral-clock model assumes that different limbs are timed by different clocks. It thus predicts that tap asynchronies will be different for the two hands after exposure to different delays, because the clocks for the left and right hand are separated and adapted separately to each specific delay (**Figure 1B**).

The hybrid-clock model assumes that there are both central and peripheral clocks, and that the peripheral clocks are linked together via the central clock. It predicts that the tap-asynchrony for the left and right hand can be recalibrated separately, but the difference will be smaller than in the multiple-clock model due to cross-talk mediated by the central clock (**Figure 1C**).

## MATERIALS AND METHODS

### PARTICIPANTS

Fifty-two participants from Kyushu Sangyo University and Tilburg University (twenty-five female, mean age 21.8, three left-handed, all using a computer mouse with their right hand) participated. Twenty-seven were assigned to a mixed-exposure condition in which the feedback delay (lag) was a within-subjects factor. The other twenty-five were assigned to a pure-exposure condition in which the feedback delay was a between-subjects factor. In the mixed-exposure condition, approximately half of the participants (fourteen) performed right-hand tapping with delayed feedback and left-hand tapping with non-delayed feedback. For the other half, the hand-delay assignment was reversed. In the pure-exposure condition, approximately half of the participants (twelve) received delayed feedback; the remaining thirteen received non-delayed feedback. All participants had normal hearing and normal or corrected-to-normal vision. Informed consent was obtained from each participant. The experiment was approved by the Local Ethics Committee of Kyushu Sangyo University and Tilburg University, and followed the declaration of Helsinki.

### STIMULI AND APPARATUS

Participants sat at a desk in a dimly lit booth looking at a white fixation cross on a CRT display (100 Hz refresh rate) at approximately 65 cm viewing distance. The auditory stimulus was a 2,000 Hz pure tone pip (30 ms duration with 2 ms rise/fall slope) presented via headphones. White noise was continuously presented via headphones to mask the sound of taps. Two special gaming mice (Logitech G300) were connected to a PC to collect the tapping data with high temporal precision (<1 ms). Participants’ hands were occluded so that they could not see the movement of their fingers.

### DESIGN

There were three factors in the experimental design. The exposure type (mixed- vs. pure-exposure) was a between-subjects factor. The test type (pre- vs. post-test) was a within-subjects factor. The feedback delay (50 ms as non-delayed vs. 150 ms as delayed) was a within-subjects factor in the mixed-exposure condition, and a between-subjects factor in the pure-exposure condition. These three factors yielded eight different experimental conditions. Each condition consisted of 20 trials.

In the mixed-exposure condition, the delay was fixed for each hand but it was different for the left and right hand. The combination of the hand (right vs. left) and the feedback delay (50 vs. 150 ms) was fixed for each participant but changed across participants. It was treated as a residual factor and was counter-balanced between participants. The order of which hand tapped first was also treated as a residual factor and was counter-balanced between participants as well.

In the pure-exposure condition, the participants were exposed to the same amount of delay (50 vs. 150 ms) for the left and right hand in the adaptation phase. The two exposure delays were run with different participants to avoid carryover effects between adaptations to different lags. The alternating order of hands was also treated as a residual factor and was counter-balanced between participants. Experimental and residual factors are summarized in **Table 1**.

**Table 1:** Experimental design and factors.

Between-subjects group	*N*	Experimental factors			Residual factors		
		Test type	Exposure type	Feedback delay	Hand for mouse-press	Hand-delay combination	Hand order
Group 1	7	Pre- and post-test (within subjects)	Mixed	Delayed (150 ms) and non-delayed (50 ms) (within subjects)	Left- and right-hand (nested in the feedback delay)	Right-hand delayed	Right first
Group 2	7						Left first
Group 3	7					Left-hand delayed	Right first
Group 4	6						Left first
Group 5	7		Pure	Non-delayed (50 ms)	Left- and right-hand (within subjects)	–	Right first
Group 6	6						Left first
Group 7	6			Delayed (150 ms)		–	Right first
Group 8	6						Left first

### PROCEDURE

**FIGURE 2:**
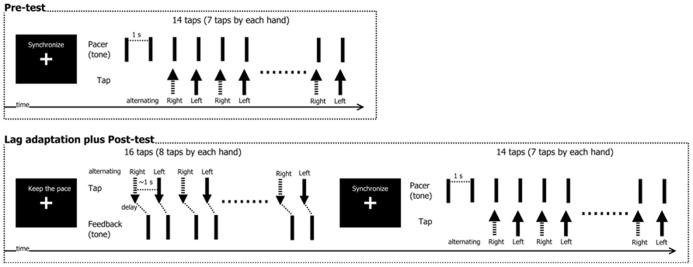
**Schematic illustration of the experimental procedure.** In the pre-test, participants tried to synchronize left-right finger taps with an isochronous tone sequence. The adaptation/post-test phase consisted of multiple short adaptation phases followed by post-tests. During adaptation, participants made *voluntary* left-right finger taps while trying to maintain a constant tempo. Each tap was followed by a feedback tone with either 50 or 150 ms delay. Following exposure to these delays, participants then again tried to synchronize their left-right finger taps with a pacer tone (as in the pre-test).

The experiment was composed of a pre-test, an adaptation phase, and a post-test (see **Figure 2**). In the pre-test, participants tried to tap (i.e., mouse-press) their left and right index fingers in synchrony with the tone that served as a pacing signal. The taps (mouse-presses) were not accompanied by any feedback signals (i.e., sounds). The tone was delivered 16 times per trial at a constant inter-stimulus interval (ISI) of 1000 ms. Participants skipped the first two pacing signals to get into the rhythm, and then synchronized their mouse presses with the following fourteen pacing signals. For each trial, there were thus seven taps for each hand.

After completion of the pre-test, the adaptation/post-test phase began. Each trial started with a short adaptation phase immediately followed by the post-test. In the adaptation phase, participants made 16 voluntary mouse-presses with their left and right index fingers in alternating order, trying to keep the inter-tap interval at approximately 1000 ms. The order (the right first, or the left first) was same as the pre-test. After each mouse-press, a tone was delivered at a constant delay at either 50 ms (non-delayed condition) or 150 ms (delayed condition), following earlier studies (e.g., Sugano et al., 2010, 2012). These values were expected to elicit quantifiable adaptive shifts, while they were still perceived as a single event (150 ms), or were expected to be perceived as subjectively simultaneous (50 ms). In the post-test that immediately followed the adaptation phase, the participants then performed the synchronization task, which was identical to the pre-test. Trials were repeated if more than two taps were missed (1.05% in total: 11 trials by 10 participants).

Participants also completed a short practice session before the experimental session in order to get acquainted with the experimental procedure. The whole experiment lasted ~50 min including the instruction, the practice session, and the experimental session.

Trials from the practice session were excluded from further analysis. The data from the pre- and the post-test were analyzed in the following analysis. Data were discarded if participants tapped on the wrong side. The tap-asynchrony was defined as the timing differences between the tap and the pacer tone, and was negative if the tap preceded the pacer. Missing responses (error taps) were only 0.48% of the total number of taps. Tap asynchronies out of the range from the mean plus minus three standard deviations (-300 to +110 ms) were regarded as outliers and were eliminated from the analysis (1.07% of the total number of taps). The first tap for each hand was also removed from the analysis because of possible instability. The rest of the tap asynchronies (six measurements per hand and per trial) were averaged over the 20 trials for each experimental condition.

## RESULTS

### AVERAGE TAP ASYNCHRONIES

The group-averaged tap asynchronies are presented in **Table 2**. All tap asynchronies were negative, which reflects the well-known anticipation tendency in sensorimotor synchronization (see, e.g., Aschersleben, 2002). The temporal recalibration effect (TRE) was defined as the change in tap-asynchrony between the pre- and the post-test. All TREs were, as expected, negative meaning that the anticipation tendency became greater after exposure to delayed and non-delayed feedback in voluntary tapping.

**Table 2:** Mean tap-asynchrony.

Exposure type	Lag (ms)	Pre-test		Post-test		Temporal recalibration effect (TRE: post – pre)	
		Mean (ms)	diff	Mean (ms)	diff	Mean (ms)	diff
Mixed-exposure	50	-79.3 (6.7)		-106.5 (5.9)		-27.2** (4.9)	
	150	-77.9 (6.3)		-124.6 (7.1)		-46.7** (5.0)	
	150 vs. 50		1.4		-18.1		-19.5
Pure-exposure	50	-92.3 (10.6)		-96.6 (9.2)		-4.3 (5.2)	
	150	-89.4 (10.8)		-133.0 (7.8)		-43.7** (5.7)	
	150 vs. 50		2.9		-36.4		-39.4

Standard errors of mean are shown in parenthesis.

**p < 0.001 (i.e., negative numbers indicate tap before pacing stimulus).

Firstly, we analyzed tap asynchronies separately for the mixed- and pure-exposure conditions. The TRE in the mixed-exposure condition was stronger for the delayed (-46.7 ms) than the non-delayed (-27.2 ms) hand. Note that there was a TRE for the non-delayed hand that possibly indicates cross-talk between the delayed and non-delayed hands. A repeated-measures ANOVA was conducted on the individual tap asynchronies in the mixed-exposure condition, with test type (pre- vs. post-test) and exposure delay (50 vs. 150 ms) as within-subjects factors. All effects were significant: test type, *F*(1,26) = 59.74, *p* < 0.001, exposure delay, *F*(1,26) = 5.63, *p* < 0.05, and the interaction between the test type × the exposure delay, *F*(1,26) = 56.99, *p* < 0.001. Separate ANOVAs per test type revealed that the effect of exposure delay was not significant in the pre-test, *F*(1,26) = 0.24, *p* = 0.63, but was significant in the post-test, *F*(1,26) = 17.20, *p* < 0.001, showing that tap-asynchrony after exposure to the delayed feedback was significantly more negative than that after the non-delayed feedback (-124.6 ms vs. -106.5 ms, respectively). To analyze the interaction between test type × exposure delay further, we used the TRE (i.e., the change between pre- and post-test) as a dependent variable and ran separate one sample *t*-tests (one-sided as there was a clear prediction) on them (with Bonferroni corrected alpha set to 0.025). As predicted, the *t*-tests showed that the TRE was significantly negative for both delayed (-27.2 ms), *t*(26) = 5.51, *p* < 0.001, and non-delayed conditions (-46.7 ms), *t*(26) = 9.39, *p* < 0.001.

Similar analyses were run in the pure-exposure conditions. The TRE was similar to the mixed-exposure condition, except that the difference between the delayed and non-delayed hand was greater in the pure-exposure (39.4 ms, ~40%) than the mixed-exposure (19.5 ms, ~20%). A mixed-model ANOVA was conducted on the individual tap asynchronies in the pure-exposure condition with test type (pre- vs. post-test) as a within-subjects factor and exposure delay (50 vs. 150 ms) as a between-subjects factor. There was a significant main effect of test type, *F*(1,23) = 36.23, *p* < 0.001, and an interaction between test type × exposure delay, *F*(1,23) = 26.02, *p* < 0.001. The main effect of the exposure delay was not significant, *F*(1,23) = 1.61, *p* = 0.22. A subsequent ANOVA for each test type with exposure delay as a between-subjects factor revealed that the effect of exposure delay was not significant in the pre-test, *F*(1,23) = 0.04, *p* = 0.85, but was significant in the post-test, *F*(1,23) = 8.94, *p* < 0.01. As with the mixed-exposure data, the TREs were entered into separate one sample *t*-tests, showing that the TRE was significantly negative for the delayed condition (-43.7 ms), *t*(11) = 7.65, *p* < 0.001, but not for the non-delayed condition (-4.3 ms), *t*(12) = 0.83, *p* = 0.21.

We also compared the TRE between mixed- versus pure-exposure by ANOVAs per exposure delay (50 vs. 150 ms) with exposure type (mixed- vs. pure-exposure) as a between-subjects factor. This ANOVA showed a main effect of exposure type (mixed- vs. pure-exposure) in the non-delayed condition (-27.2 ms vs. -4.3 ms for mixed- vs. pure-exposure, respectively), *F*(1,38) = 8.19, *p* < 0.01, while it was not significant in the delayed condition (-46.7 ms vs. -43.7 ms for mixed- vs. pure-exposure, respectively), *F*(1,37) = 0.13, *p* = 0.72. In mixed-exposure, the delayed hand thus affected the non-delayed hand, but not *vice versa*.

### BUILD-UP OF TR

**FIGURE 3:**
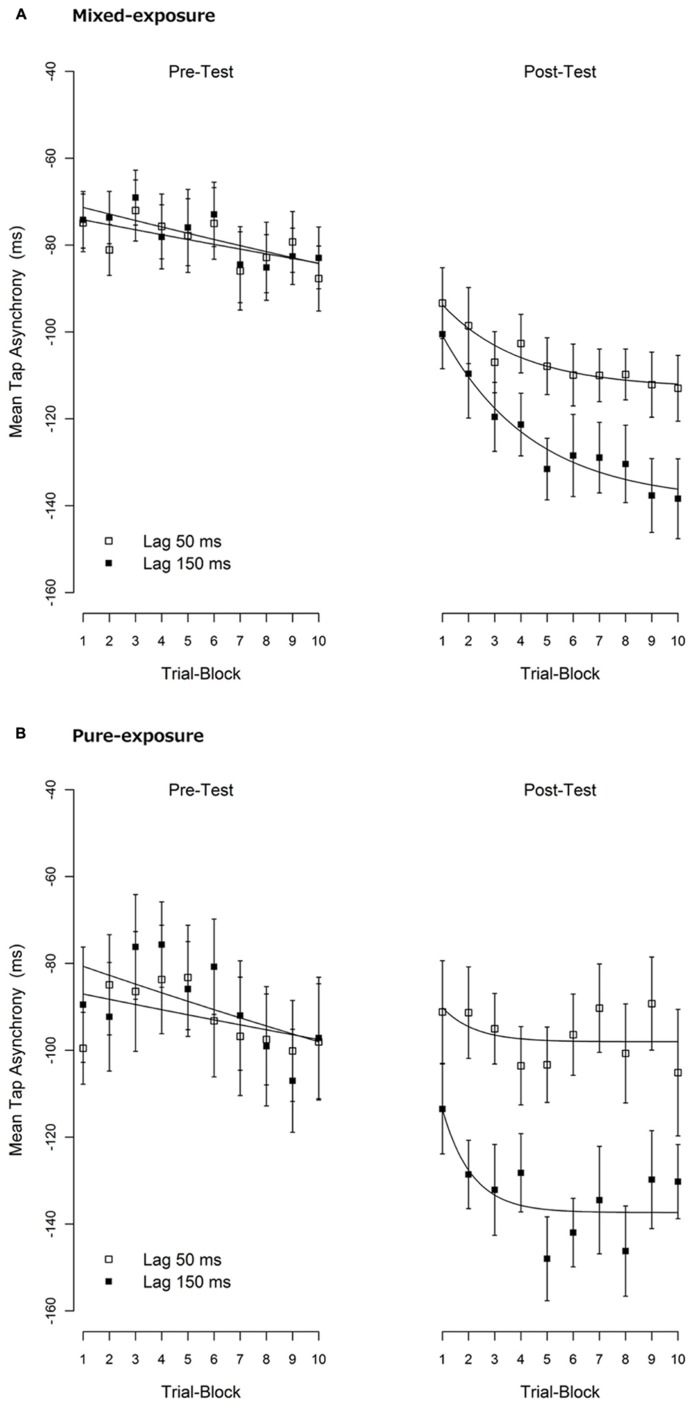
**Mean tap asynchronies per trial-block. (A)** The mixed-exposure condition. **(B)** The pure-exposure condition. One trial-block contains two consecutive trials. A negative tap-asynchrony means that the tap comes before the tone (i.e., an anticipation error). Error bars represent 1 standard error of mean (SEM). An exponential decay function, *P*_2_ +(*P*_0_ - *P*_2_ ) × exp (-*P*_1_ × x), was fitted to the mean tap asynchronies over the trial-blocks. The meaning of each parameter was explained in the text. The fitted lines are shown in solid lines.

Secondary analyses were performed to examine the build-up of the TRE. To examine this, we divided the 20 trials of each condition into 10 blocks of two trials each. The mean tap asynchronies per block are shown in **Figure 3**.

An exponential decay function, *P*_2_ + (*P*_0_ - *P*_2_ ) × exp (-*P*_1_ × x), was fitted to the mean tap asynchronies over the trial-blocks where P_0_ reflects a “starting point” before adaptation (x=0), P_1_ reflects a “rate of change” (the greater, the faster the decay) and P_2_ reflects an “end point” after adaptation was completed (x → ∞ ). The fitting was carried out using the NLS function in the statistical package R version 2.12.1 (R Development Core Team, 2010) with the NL2SOL algorithm, which gave the non-linear least-squares estimates of fitting parameters. The fitted lines are shown in **Figure 3**, and the estimated values of the parameters are shown in **Table 3**.

**Table 3:** Estimated parameters in fitting a decay function for the mean tap asynchronies.

Exposure type	Lag (ms)	Pre-test			Post-test		
		P_0_ (Starting point)	P_1_ (Rate of change)	P_2_(End point)	P_0_(Starting point)	P_1_(Rate of change)	P_2_(End point)
Mixed-exposure	50	-73.0 (5.2)	0.010 (0.344)	-187.7 (3.7x10^3^)	-86.1 (4.4)	0.334 (0.113)	-113.0 (2.1)
	150	-69.8 (4.8)	0.013 (0.242)	-185.2 (1.9x10^3^)	-87.9 (6.1)	0.289 (0.082)	-139.0 (3.9)
Pure-exposure	50	-85.8 (8.2)	0.011 (0.508)	-200.2 (5.1x10^3^)	-80.2 (34.8)	0.816 (1.612)	-98.1 (2.9)
	150	-78.6 (11.3)	0.016 (0.421)	-207.0 (3.0x10^3^)	-79.5 (48.6)	0.890 (0.713)	-137.4 (3.3)

*Standard errors are shown in parenthesis.*

*Note: the decay function is, f(x)=P_2_ + (P_0_ - P_2_ ) × exp (-P_1_ × x)
*

As can be seen in **Figure 3**, although the mean tap-asynchrony in the pre-test slowly declined across trial-blocks, the trends were almost the same across experimental conditions confirming that the baseline was the same across conditions. A possible reason for the gradual increment of the asynchrony in the pre-test might be a reduced tactile sensitivity (due to a fatigue of mechano-receptors or a decrease of attention for the tactile feedback) caused by repeated tapping. If tactile sensitivity declines, then the latency of the tactile feedback might increase, thus causing a bigger tap-asynchrony (e.g., Aschersleben et al., 2001).

The mean tap-asynchrony of the delayed condition in the post-test sharply declined and quickly reached a plateau in the pure-exposure condition when compared with the mixed-exposure condition. This observation was supported by the fact that the estimated parameter reflecting “rate of change” (P_1_ ) was greater in the pure than the mixed-exposure (0.890 vs. 0.289, respectively), albeit not significantly different, *t*(16) = 0.84, *p* = 0.41. The P_1_ parameter of the non-delayed condition showed the same pattern between the pure- and mixed-exposure (0.816 vs. 0.334, respectively), though the difference was again not significant, *t*(16) = 0.30, *p* = 0.77. The reason why we could not get significant P_1_ differences in the post-test is, at least in part, due to a relative larger standard error in estimating P_1_ in the pure-exposure condition than in the mixed-exposure condition (1.612 and 0.713 vs. 0.113 and 0.082, see **Table 2**). These results suggest that the build-up of the TRE tended to be slower and less complete in the mixed-exposure condition than in the pure-exposure condition.

### DISSIPATION OF TR

**FIGURE 4:**
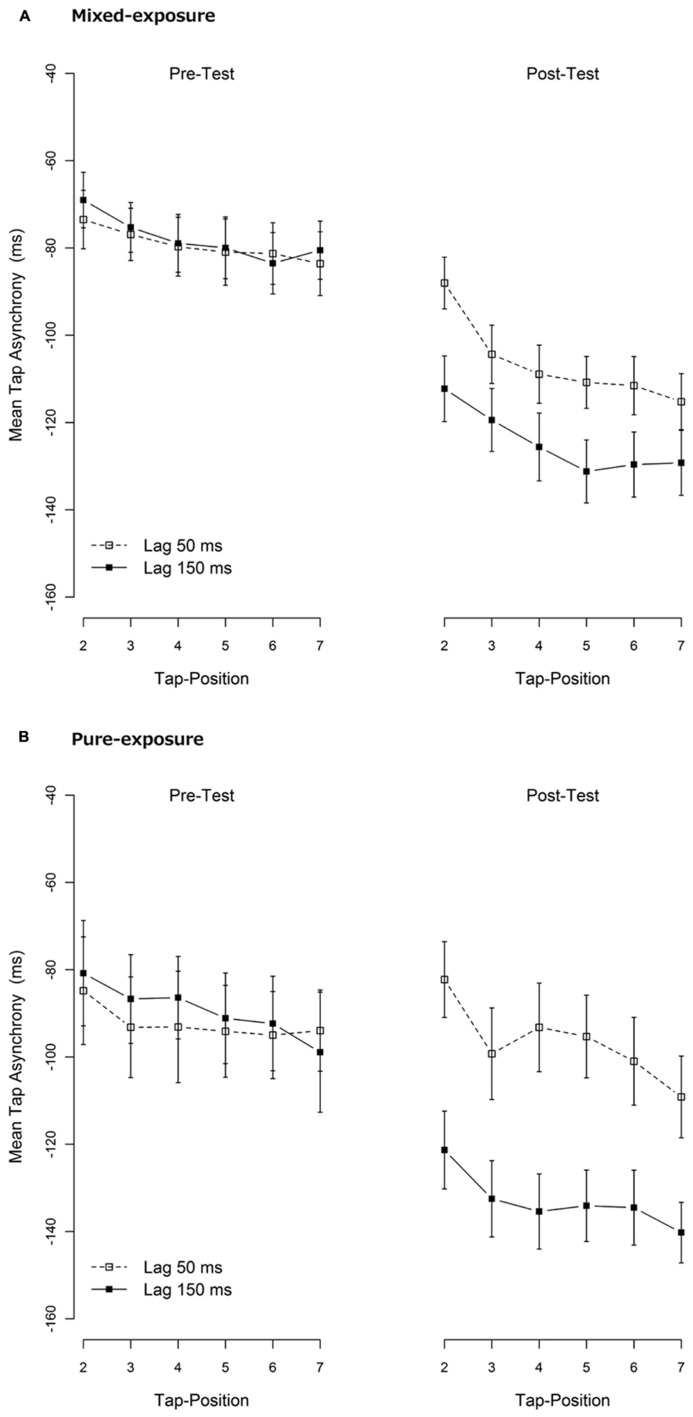
**Mean tap asynchronies per tap. (A)** The mixed-exposure condition. **(B)** The pure-exposure condition. They were re-calculated from the same data as shown in **Figure 3**. Data of the first tap-position were omitted due to instability. A negative tap-asynchrony means that the tap comes before the tone (i.e., an anticipation error). Error bars represent 1 standard error of mean (SEM).

To examine if there was dissipation of TR, mean tap asynchronies for each tap within a trial were calculated. The mean tap asynchronies across hands for the 2nd until the 7th tap (1st tap was omitted from the analysis as mentioned before) are shown in **Figure 4**. As is clearly visible, although the tap asynchronies in the post-test became more negative as the number of taps increase, the difference between the delayed and the non-delayed conditions remained constant in all taps. To confirm this, mean tap asynchronies per tap were entered into a repeated-measures or mixed-model ANOVA per exposure type (mixed- vs. pure-exposure) and test type (pre- vs. post-test), with tap position (2nd to 7th tap) as a within-subjects factor and exposure delay (50 vs. 150 ms) either as a within-subjects (mixed-exposure) or a between-subjects factor (pure-exposure). As expected and shown in **Figure 4**, these ANOVAs revealed a significant main effect of tap position (in the pre- and post-test) and exposure lag (in the post-test only) under both exposure types (all ps < 0.05). Most importantly, the tap position did not interact with exposure delay in either exposure type in the post-test, *F*(5,130) = 1.71, *p* = 0.14, in mixed-exposure and *F*(5,115) = 0.84, *p* = 0.53, in pure-exposure, indicating that the TRE did not dissipate within the short term period of one trial (~14 s).

### VARIABILITY OF TAP ASYNCHRONIES

Similar analyses were conducted on the variability (the standard deviation) of the tapping responses. The group-averaged standard deviations are shown in **Table 4**.

**Table 4:** Mean standard deviation of tap-asynchrony.

Exposure type	Lag (ms)	Pre-test		Post-test		Post – Pre
		Mean SD (ms)	diff	Mean SD (ms)	diff	Mean SD (ms)	Diff
Mixed-exposure	50	42.9 (1.6)		50.8 (2.7)		7.9 (1.8)	
	150	44.2 (1.8)		50.0 (3.1)		5.8 (2.0)	
	150 & 50	43.5	1.3	50.4	-0.8	6.9**	-2.1
Pure-exposure	50	53.2 (3.1)		57.9 (3.1)		4.7 (2.0)	
	150	52.0 (3.7)		55.4 (3.8)		3.5 (2.1)	
	150 & 50	52.6	-1.2	56.7	-2.5	4.1*	-1.2

Standard errors of mean are shown in parenthesis.

*p < 0.01, **p < 0.001.

A repeated-measures and a mixed-model ANOVA were applied separately for the mixed- and the pure-exposure conditions respectively, with test type as a within-subjects factor and exposure delay as a within-subjects (mixed-exposure) or a between-subjects factor (pure-exposure). ANOVAs showed that only a main effect of test-type (pre- vs. post-test) was significant in both exposure types, *F*(1,26) = 15.45, *p* < 0.001 (mixed-exposure), *F*(1,23) = 8.16, *p* < 0.01 (pure-exposure), showing that the variability of taps became greater (less stable) in the post-test (50.4 ms for the mixed-exposure, 56.7 ms for the pure-exposure) than in the pre-test (43.5 ms for the mixed-exposure, 52.6 ms for the pure-exposure). The variability of tapping after an exposure to delayed sensory feedback was comparable to that after non-delayed feedback, suggesting that the TR occurs without changing the stability of tapping.

## DISCUSSION

In the present study, we tested whether concurrent TR for different feedback delays is possible in sensorimotor coordination (finger tapping). During a short adaptation phase, participants tapped their left and right fingers in alternating fashion while a tone was delivered 50 ms (a subjective no-delay) or 150 ms (delayed) after each tap. After this adaptation phase, participants then tried to tap in synchrony with pacing tones. In line with previous studies (Sugano et al., 2012), results showed that the tap-asynchrony became greater (i.e., a larger anticipation error) after exposure to delayed feedback, presumably because participants shifted their motor timing or the perceived timing of the sensory signal to compensate for the delay (i.e., a temporal recalibration effect: TRE). Importantly, when the left and right hands were concurrently exposed to different delays, then each hand displayed a different amount of TRE. This means that concurrent TR for different delays is possible in the sensorimotor domain, as it is in the audio-visual domain (Roseboom and Arnold, 2011; Heron et al., 2012; Yuan et al., 2012).

It is also of note that the non-delayed hand in the mixed-exposure condition increased its anticipation error, but not so in the pure-exposure condition. This suggests that there might be a cross-talk of TRE between hands in mixed-exposure. Further evidence for cross-talk can be found in the build-up course of the TRE, which tended to be slower and less complete in mixed than in pure-exposure.

The results of the present study argue in favor of a motor-shift account for sensorimotor TR, which assumes that it is the motor timing (e.g., when did I move my finger or when did my finger hit the pad?) rather than sensory timing (e.g., when did I hear the sound?) which is recalibrated with delayed sensory feedback, as the left and right hand can be recalibrated differently. Earlier research also suggested that TR of sensorimotor events is mainly caused by a shift in the motor component (Sugano et al., 2010, 2012, Stekelenburg et al., 2011). However, when discussing the motor or sensory nature of TR, it is important to be cautious because motor timing is not a single entity, rather it can be decomposed into several components such as an intention to move, an actual motor command, an efferent copy of that command, a proprioceptive feedback about the movement and the position of the joints, and tactile feedback from clicking the mouse (e.g., Frissen et al., 2012; Sugano et al., 2012). At present, it is difficult to elucidate which component actually has been adjusted by TR because all these components are correlated. To disentangle them, future research might measure the timing of various action-related components. One approach might use the Libet clock-hand paradigm (Libet et al., 1983) to measure the timing of the intention to move.

### POSSIBLE MECHANISM FOR CONCURRENT ADAPTATION

The present results are most easily accounted for by a hybrid-clock model in which a single central clock and multiple peripheral clocks are linked together (**Figure 1C**). This model is closely related with a two-level model for motor timing in which a timing goal is represented at a central level and a movement itself is generated by an automatic motor system (e.g., Semjen and Ivry, 2001). In this view, the left and right hands have their own peripheral clocks that are linked via a central clock. The peripheral clocks are in charge of sensorimotor timing for the left and right hand independently. The central clock is a master clock that regulates a global timing goal and links multiple peripheral clocks. This hybrid-clock model predicts that the left and right hand can be recalibrated differently, though its size should be smaller than the prediction generated by the multiple-clock model.

The single-central-clock model and the multiple-peripheral-clock model do not predict the present results well. The former assumes that a single central clock regulates all the timing in the brain and predicts that the left and the right hand are recalibrated the same way (**Figure 1A**). The latter assumes that different limbs are timed by different clocks and predicts that the two hands are recalibrated independently after exposure to different delays (**Figure 1B**).

It has been suggested that the mechanism of interval timing is separated into two systems, a cognitively controlled one and an automatic one (Lewis and Miall, 2003; Buhusi and Meck, 2005; Repp and Su, 2013). The central clock component might correspond to the cognitively controlled timing mechanism, whilst the multiple peripheral components might correspond to the automatic timing mechanism. It is of importance to realize that the time scale in which these two distinct timing systems work is different. The cognitively controlled mechanism works mainly in the supra-second range, whilst the automatic system works in a sub-second range (Lewis and Miall, 2003; Buhusi and Meck, 2005; Repp and Su, 2013). In line with this dichotomy, motor timing is thought to be controlled by the automatic system that works in a sub-second range.

Another well-known dichotomy in timing control of rhythmic finger tapping is the difference between phase correction (i.e., adjustment of a tap-stimulus synchronization) and period correction (i.e., adjustment of inter-tap intervals; Mates, 1994a,b; Repp, 2001a,b). They differ in their degrees of cognitive control and may be associated with different brain circuits (Repp, 2005). Period correction may need more cognitive control than phase correction, which is largely automatic (Repp, 2005). The peripheral clocks might be more related with phase correction while the central clock might be engaged in period correction. If so, then more cross-talk of TRE might be observed in a synchronization task with tempo changing pacing signals such as gradual tempo acceleration and/or deceleration because tuning-in the tempo requires a cognitively controlled period correction mechanism (e.g., Repp, 2005; Schulze et al., 2005).

### NEURAL CORRELATES OF CENTRAL AND PERIPHERAL CLOCKS

What neural mechanisms or regions are candidates for the centralized single clock and the peripheral localized clocks? The cerebellum and the thalamo-cortico-striatal circuits might be candidates for the peripheral and the central timing mechanism, respectively.

The automatic timing system is thought to be controlled mainly by the cerebellum. Dysfunction of cerebellum causes impairments in synchronization (Repp and Su, 2013) and motor adaptation (Bastian, 2008). It has been shown that patients with a unilateral deficit in the cerebellum showed impaired tapping only for the impaired, ipsilateral side (Ivry et al., 1988), suggesting that the cerebellar hemispheres have a separate clock controlling the sensorimotor synchronization task for each side. Moreover, activation of the cerebellum is context-dependent, thus suggesting localized, rather than centralized representation of time (Coull and Nobre, 2008). Although the cerebellar hemispheres might be interconnected during bimanual tapping (Pollok et al., 2005b), this might not be the case during the unimanual tapping (Pollok et al., 2005a). The alternate tapping by either hand, as used in the present study, is thought to utilize a similar timing control mechanism as the one in unimanual tapping (Summers et al., 1989; Semjen and Ivry, 2001). Accordingly, the left and right cerebellar hemispheres might be working in isolation during the adaptation phase. The cerebellum might thus be a possible candidate for the peripheral timing control to different feedback delays.

The thalamo-cortico-striatal circuits are thought to be involved in the cognitively controlled timing system which works in a supra-second range (Buhusi and Meck, 2005) and are subject to attentional modulation (Repp and Su, 2013). In the thalamo-cortico-striatal network, it has been shown that the left dorsal premotor cortex (dPMC) is crucial for accurate timing of either hand (Pollok et al., 2008; Bijsterbosch et al., 2011). More generally, the left hemisphere dominates over both the left- and right-hand tapping (Pollok et al., 2005b, 2008), irrespective of hand dominance (Pollok et al., 2006). Activity of the basal ganglia has also been shown to be independent of the motor effectors (right/left hand, speech) used in rhythmic timing (Bengtsson et al., 2005). Pollok et al. (2005b) found a coupling between the left and right premotor areas during bimanual tapping, which led them to suggest that a cross-talk between the limbs might occur at the level of motor planning and programming. Furthermore, recent studies have shown that there is nearly perfect intermanual transfer of various motor-skills including visual-motor learning (Imamizu and Shimojo, 1995), anticipatory timing (Teixeira, 2000), motor-skill learning (Perez et al., 2007) and a pegboard task (Schulze et al., 2002), suggesting that there is cross-talk between the hemispheres in the intermanual transfer of motor learning. The cerebral hemispheres, especially the PMC and the supplementary motor area (SMA), are probably the crucial loci for it (e.g., Schulze et al., 2002; Perez et al., 2007; Kirsch and Hoffmann, 2010). Possibly, then, cross-talk of TR between hands might occur in these higher cortical networks.

### WHY WAS TR OF THE DELAYED HAND IN MIXED-EXPOSURE SIMILAR TO PURE-EXPOSURE?

One result that is somewhat difficult to reconcile with a cross-talk account is that the TRE of the delayed hand reached the same level in mixed as in pure-exposure, whereas a cross-talk account would predict a smaller effect in mixed-exposure. One speculation is that the 150 ms delay in the mixed-exposure condition became more noticeable due to a contrast effect. Although somewhat anecdotic, several participants in the mixed-exposure condition reported that they noticed the 150 ms delay, whereas this was rarely the case in pure-exposure. Possibly, the staggered pattern of delays after the right- and left-hand tapping might have participants attend to the delayed feedback itself instead of the task-relevant inter-tap intervals. It has been reported that attention to delayed timing can boost TR in the audio-visual domain (Heron et al., 2010), and possibly a similar mechanism works in the sensorimotor domain, thus boosting the TR for the delayed hand in the mixed-exposure condition.

## CONCLUSION

This study demonstrates that tapping in synchrony with a pacer tone can be used as a viable measure of TR. It appears that TR has both central and motor-specific components (see also, Sugano et al., 2012). The timing of the left and right hand could be adjusted differently after exposure to different delays of sensory feedback. This concurrent adaptation to different delays occurred slower and was less complete than when both hands were exposed to the same delay, thus suggesting that there was cross-talk of adaptation between the hands. These results are best explained by a hybrid-clock model with linked central and peripheral internal time-keepers.

## AUTHOR CONTRIBUTIONS

Yoshimori Sugano, Mirjam Keetels, and Jean Vroomen designed the research; Yoshimori Sugano and Mirjam Keetels performed the experiment; Yoshimori Sugano analyzed data; and Yoshimori Sugano, Mirjam Keetels, and Jean Vroomen wrote the paper.

## Conflict of Interest Statement

The authors declare that the research was conducted in the absence of any commercial or financial relationships that could be construed as a potential conflict of interest.
